# Social Isolation-Mediated Exacerbation of Negative Affect in Young Drinkers during the COVID-19 Pandemic

**DOI:** 10.3390/brainsci12020214

**Published:** 2022-02-04

**Authors:** Gopi K. Neppala, Isabel Terkuhle, Ariella Wagner, Lauren Lepow, Riaz B. Shaik, Rachel Freed, David Kimhy, Robert H. Pietrzak, Eva Velthorst, Adriana Feder, Iliyan Ivanov, Muhammad A. Parvaz

**Affiliations:** 1 Department of Psychiatry, Icahn School of Medicine at Mount Sinai, New York, NY 10029, USA; gopi.neppala@icahn.mssm.edu (G.K.N.); it2293@tc.columbia.edu (I.T.); ariella.wagner@mssm.edu (A.W.); lauren.lepow@icahn.mssm.edu (L.L.); riaz.shaik@mssm.edu (R.B.S.); rachel.freed@mssm.edu (R.F.); david.kimhy@mssm.edu (D.K.); eva.velthorst@mssm.edu (E.V.); adriana.feder@mssm.edu (A.F.); iliyan.ivanov@mssm.edu (I.I.); 2 U.S. Department of Veterans Affairs, National Center for Posttraumatic Stress Disorder, VA Connecticut Healthcare System, West Haven, CT 06516, USA; rhpietrzak@gmail.com; 3 Department of Psychiatry, Yale School of Medicine, New Haven, CT 06511, USA; 4 Department of Social and Behavioral Sciences, Yale School of Public Health, New Haven, CT 06520, USA; 5 Department of Neuroscience, Icahn School of Medicine at Mount Sinai, New York, NY 10029, USA

**Keywords:** COVID-19, adolescents, alcohol use, stress, affect, resilience, impulsivity, anhedonia

## Abstract

Emerging research on psychological adjustment during the COVID-19 outbreak has suggested that young people may be particularly vulnerable to increases in negative affect during the pandemic. However, the association between alcohol use in youth and change in negative affect during this unprecedented time is not clear. Using an online survey, this study obtained scores on negative affect (*before* and *during* the COVID-19 pandemic), pandemic-related stress, change in drinking frequency, and traits including resilience, impulsivity and anhedonia, from a sample of drinkers and non-drinkers, up to the age of 21. Young drinkers experienced a greater increase in negative affect during the pandemic compared to non-drinkers, and this differential rise in negative affect was mediated by the pandemic-related stress of social isolation. Young drinkers also experienced a decrease in alcohol use during the pandemic, but this was not associated with a change in negative affect. Interestingly, young drinkers with greater resilience and lower anhedonia reported less increase in negative affect during the COVID-19 pandemic. Taken together, these results show that the greater increase in negative affect that young drinkers experienced during the COVID-19 pandemic, compared to their non-drinking counterparts, was mediated by pandemic-related social isolation. Moreover, greater resilience and lower anhedonia may have served as protective factors for mitigating the social isolation-induced worsening of negative affect in young drinkers during the pandemic. These findings may inform future studies investigating potential indicators of maladaptive affective responses to public health crises in vulnerable adolescent populations.

## 1. Introduction

At the onset of the COVID-19 pandemic, one of the first responses to contain and reduce the exponentially growing infection and death rates was to implement a lockdown, with severely limited in-person social exposure. Only recently have studies on the mental health impacts of pandemic-related social isolation among young people started to emerge [1]. Social isolation has been shown to exacerbate pre-existing anxiety and depression, which are both highly comorbid, with harmful drinking in emerging adulthood [2,3]. Studies have begun to point to the detrimental effects of COVID-19 on mental health, including increased symptoms associated with depression, perceived stress, and alcohol use [4]. However, the impact of pandemic-related social isolation on the mental health of young drinkers, who typically procure alcohol through their social contacts [5], is currently not known.

Underage drinking is a major societal concern; it has been estimated that adults aged 26 and older, who initiated alcohol use at age 15 or younger, were 5.6 times more likely to report an alcohol use disorder in the past year than those who began drinking at age 21 or older [6]. Alcohol use is most commonly initiated during adolescence, which also marks a critical period for neurobiological maturation, posing serious risks to healthy brain development, especially the prefrontal cortex, responsible for task execution, judgement, reasoning, planning, and problem solving, increasing the risk of developing psychiatric problems in adulthood [7,8,9]. Negative affect, such as anger and fear, has been shown to be associated with alcohol use during adolescence [10]. Furthermore, negative affect has been shown to statistically predict substance use, including alcohol drinking in adolescents over time [11], and other studies have suggested that drinking to cope may in turn lead to worsened negative affect [12].

The stress from social isolation, during the COVID-19 pandemic specifically, may impact negative affect levels among adolescents, especially for those with a history of alcohol use. Social isolation often contributes to and maintains alcohol use behaviors, and problematic alcohol users tend to experience greater social isolation than non-users [13,14]. In adults, emerging studies have suggested that harmful alcohol use increased during the COVID-19 pandemic for individuals under lockdown orders, when compared with individuals not under lockdown [15]. Recent research has already pointed to the detrimental effects of pandemic-induced social isolation on affective cognitive processes in adults [16]. Further, according to a recent systematic review of 80 studies on adolescent mental health, young people experience the effects of loneliness and social isolation more acutely than adults, which in turn increases the risk of depression among youths [17]. These findings demonstrate the established links that social isolation has with both alcohol use and negative affect, particularly among young people. Considering that social isolation has been a hallmark of the current public health crisis [18], there is a clear need to investigate whether the greater stress resulting from social isolation plays a role in mediating a relationship between early drinking and increased negative affect during the pandemic.

Several risk and protective factors might also play key roles in the relationship between early drinking and worsening negative affect during the COVID-19 pandemic, including resilience, impulsivity, and anhedonia. Resilience, which is often defined as “an individual’s ability to thrive in the face of adversity” [19], has been identified as a moderator between stress and alcohol dependence, as well as a protective factor against the impact of stress on negative emotions [20]. Although greater resilience has been shown to be correlated with higher levels of life satisfaction, lower depressive symptoms, and a reduced risk of alcohol use disorder in adolescents [21,22], its interaction with a stress-induced increase in negative affect in alcohol-consuming youth has not been examined. Trait impulsivity is defined as a predisposition towards rash and unplanned reactions to stimuli without regard for negative consequences [23] and has been demonstrated to have clear links to substance and alcohol use behaviors [24]. Studies have also suggested that negative affect may be deleterious to short-term impulse control and decision-making among young drinkers [25]. Anhedonia, defined as the inability to enjoy previously pleasurable activities [26], is another factor that has been associated with stress reactivity [27,28], alcohol use [29] and negative affect [30]. In adolescents, recent studies have shown a link between anhedonia and stress-induced negative affect, such that stressful situations lead to greater negative affect in youths with greater anhedonia [31,32]. Indeed, the established link between negative affective states, alcohol use, and these three factors (resilience, impulsivity, and anhedonia) justifies further examination of the interaction between these factors and changes in negative affect in young alcohol users, in the context of social isolation during COVID-19 lockdown.

Thus, the purpose of this study was to examine the following: (1) whether regular alcohol use prior to the COVID-19 pandemic renders young drinkers vulnerable to increased negative affect; (2) whether the pandemic-related stress of social isolation influences the relationship between early alcohol use and a change in negative affect; and (3) whether traits such as resilience, impulsivity, and anhedonia are differentially associated with a change in negative affect between alcohol drinking youths and their non-drinking counterparts.

## 2. Methods

### 2.1. Participants

Participants (at least 12 years old) were recruited through social media advertisements and consented to participate in this study, which was approved by the Institutional Review Board of the Icahn School of Medicine at Mount Sinai. The study sample included 220 participants from within the United States, aged 21 and younger (Table 1), who completed an online survey study through the Research Electronic Data Capture (REDCap) platform. This survey recorded data from 2 July 2020, to 14 January 2021, during which time public schools in most of the United States were closed for in-person learning and non-essential workers were advised to stay home. The study comprised mostly of females (*n* = 161, 73.5%). Almost half of our participants were 18 years old or younger (*n* = 108, 49.1%). A majority of participants also reported having received a mental health-related diagnosis by a clinician (*n* = 139, 63.2%), of whom 116 reported “Anxiety” or “GAD” (generalized anxiety disorder), 91 reported “depression” or a “depressive disorder”, 25 reported “OCD” (obsessive-compulsive disorder), and 22 reported “ADHD” (attention-deficit/hyperactivity disorder). Of the 220 participants, 162 reported no or rare use of alcohol *before* the pandemic, 30 participants reported drinking once or several times a month, and 28 participants reported drinking at least once a week. Participants were grouped as drinkers (*n* = 58; who reported drinking at least once a month) or non-drinkers (*n* = 162; who reported no or rare alcohol use) based on their alcohol use *before* the pandemic from their responses to the Coronavirus Health Impact Survey (CRISIS) [33] administered in this study (see Section 2.2 below).

### 2.2. Measures

Coronavirus Health Impact Survey (CRISIS): The CRISIS survey, administered between July 2020 and February 2021, was used to evaluate the impact of the COVID-19 pandemic on the mental health and behavior of study participants. The survey includes the following seven domains: demographics, health and exposure status to COVID-19; life changes due to the COVID-19 pandemic (e.g., changes in employment or schooling); behaviors (e.g., sleep, time spent outdoors); emotions (e.g., worry, fear); media use (e.g., social media, video games); and substance use (e.g., alcohol, marijuana). The latter four domains asked participants to provide responses on a Likert scale based on both *before* (3 months prior to the COVID-19 related stay-at-home restrictions being instituted for the participant) and *during* (since the COVID-19 related stay-at-home restrictions were instituted for the participant) the pandemic [33]. Responses to five questions from the emotions domain of the CRISIS survey, asking participants to report their levels of worry, fear, jitteriness, fatigue, and irritability, both *before* and *during* the pandemic, on a five-point Likert scale from 1 (“not at all”) to 5 (“extremely”), were averaged to yield a composite measure of negative affect, modeled after scoring of the Positive and Negative Affect Schedule (PANAS) [34]. The CRISIS survey also asked participants to report their frequency of alcohol use on a 1 (“not at all”) to 8 (“more than once a day”) scale, which as described previously, was used to categorize participants as either drinkers or non-drinkers. Additionally, the CRISIS survey also assessed frequency of marijuana and tobacco use using this same scale [33].

Connor–Davidson Resilience Scale (abbreviated version, CD-RISC2): The CD-RISC2 is a validated 2-item version of the 20-item CD-RISC for measuring resilience and its total score has demonstrated a significant correlation with each item on the original 20-item scale (*p* < 0.0001 and r = 0.27 to 0.66) [35]. These two items asked if participants are able to “adapt when changes occur” and if they are “easily discouraged by failure” on a scale from 0 (“not true at all”) to 4 (“true nearly all the time”). The computed total of responses to these two questions was used as the score for this survey.

Negative Urgency, Premeditation (lack of), Perseverance (lack of), Sensation Seeking, Positive Urgency, Impulsive Behavior Scale (abbreviated version, S-UPPS-P): The S-UPPS-P is a validated 20-item version of the 59-item UPPS-P questionnaire that is used to evaluate impulsivity by measuring the five listed impulsive traits (negative urgency, positive urgency, lack of premeditation, perseverance, and sensation seeking). Items were scored on a Likert scale from 1 (“agree strongly”) to 4 (“disagree strongly”). S-UPPS-P has also demonstrated an internal consistency with the original 59-item survey (α = 0.74 to 0.88 across subscales). As done previously, the additive total score was used in this study, with higher scores indicating greater trait impulsivity [36].

Temporal Experience of Pleasure Scale (TEPS): The TEPS is a validated 18-item questionnaire used to evaluate anhedonia by measuring anticipatory and consummatory facets of pleasure. The total score on the TEPS shows a strong internal consistency (α = 0.78). Items were scored on a Likert scale from 0 (“very false for me”) to 5 (“very true for me”). Only the total score on this survey was considered for this analysis. Higher scores on this survey indicated a greater capacity to experience pleasure, and conversely, lower anhedonia [37].

### 2.3. Data Analysis

Change in negative affect was analyzed using a 2 × 2 mixed ANOVA, with Time (*before* and *during* the pandemic) as the within-group factor and Alcohol Use (drinkers vs. non-drinkers) as the between-group factor. The dependent variable was the composite Negative Affect measure, which was calculated by averaging each participant’s ratings of the five emotions (worry, fear, jitteriness, fatigue, irritability) from the emotions domain of the CRISIS survey, separately for *before* and *during* the pandemic. Variables from the Demographics domain of the CRISIS survey that were both significantly different between groups (assessed via chi-square test for categorical variables and Wilcoxon rank-sum test for continuous variables) and were significantly associated with change in negative affect over time (assessed via univariate ANOVAs for categorical variables and Spearman rank correlations for continuous variables) were included as covariates. To examine whether the effects are specific to alcohol use, we included the frequencies of marijuana and tobacco use *before* the pandemic as covariates in the ANOVA. Negative affect ratings, either *before* or *during* the pandemic, that were either greater than 1.5 times the interquartile range, above the 3rd quartile, or lower than 1.5 times the interquartile range below the 1st quartile were considered as outliers and were removed from our analysis. Statistically significant Time × Alcohol Use interaction from the mixed ANOVA was followed by a pairwise comparison for estimated marginal means of negative affect scores at both time points between drinkers and non-drinkers, while controlling for any covariate(s) originally included in the mixed ANOVA.

Change in alcohol use among drinkers and non-drinkers during the pandemic, based on the CRISIS survey, was also assessed using separate within-group Wilcoxon signed-rank tests between alcohol use *before* and *during* the pandemic. Additionally, Spearman rank correlations among drinkers were conducted to measure for an association between change in negative affect (*during* minus *before* scores) and (1) frequency of alcohol both *before* and *during* the pandemic and (2) change in alcohol use (*during* minus *before* scores).

Mediation analyses were conducted to explore whether the relationship between alcohol use and pandemic-related change in negative affect, if found to be significant on the mixed ANOVA, is mediated by the pandemic-related stress of social isolation. From the CRISIS survey, we identified the following three questions that measured social isolation-related stress: “How stressful were the restrictions on leaving home for you?”, “How stressful were the changes in social contacts for you?” and “How stressful were the changes in family contacts for you?”. These questions were all asked on a five-point Likert scale and referred to *during* the pandemic. Answers that were significantly different between the two groups were used to test for mediation between drinking and change in negative affect. These mediation analyses were conducted using bootstrapping procedures with unstandardized effects calculated for each of 1000 bootstrapped samples. Alcohol Use served as the independent variable (0 = non-drinkers, 1 = drinkers) and the change in pandemic-related Negative Affect (*during* minus *before* scores) as our dependent variable. Mediation was considered significant only if the indirect effect of the potential mediator reached statistical significance (*p* < 0.05).

Spearman correlation tests were conducted between change in Negative Affect and total scores for CD-RISC2, S-UPPS-P and TEPS within all participants and separately within drinkers and non-drinkers. Considering the observed response drop off between individuals that completed CRISIS (*n* = 220) and these three surveys (CD-RISC2 (resilience, *n* = 125), S-UPPS-P (impulsivity, *n* = 102), and TEPS (anhedonia, *n* = 73)), we elected to analyze the results of CD-RISC2, S-UPPS-P, and TEPS separately to maximize statistical power. Fisher’s z-transformation was used to determine if correlation coefficients significantly differed between drinkers and non-drinkers. All analyses in this study were completed using *R* software.

## 3. Results

### 3.1. Change in Negative Affect

**Figure 1:**
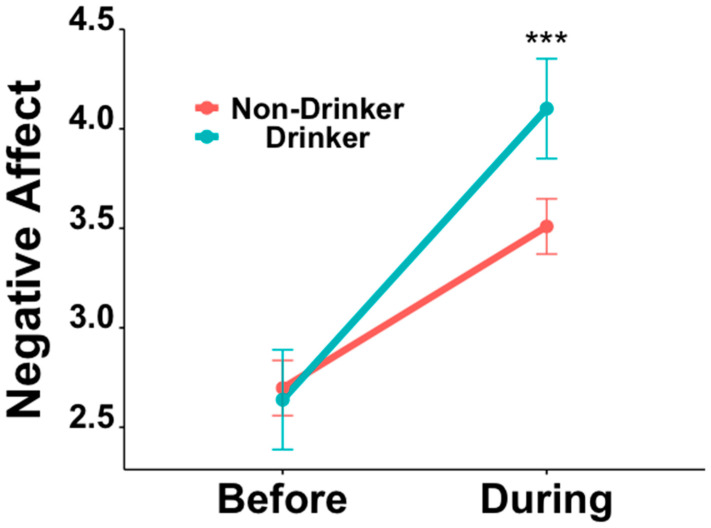
ANCOVA comparing the change in Negative Affect scores from *before* to *during* the pandemic between young drinkers and non-drinkers. *** indicates a significant difference in negative affect scores at said time point based on our pairwise comparison.

Negative Affect scores from three drinkers were deemed outliers and, therefore, removed from all of our analyses (final sample analyzed: drinkers (*n* = 55); non-drinkers (*n* = 162)). Two non-drinkers were also removed from this analysis on change in Negative Affect, specifically because they did not report their marijuana use. The highest level of education and age group were included as covariates in the ANOVA, as these variables were significantly different between the two groups (Education: drinkers > non-drinkers, Z = −6.40, *p* < 0.001; Age: drinkers > non-drinkers, Z = −7.34, *p* < 0.001) and correlated with change in Negative Affect over time (Education: r = 0.195, *p* = 0.004; Age: r = 0.211, *p* = 0.002). Additionally, the frequencies of marijuana and tobacco use *before* the pandemic were also added as covariates to study effects that are specific to alcohol use. The 2 × 2 ANCOVA on Negative Affect, controlling for the frequency of marijuana use, frequency of tobacco use, highest level of education, and age, showed the expected main effects of Time (*during* > *before*; F = 12.817, *p* < 0.001, df = 209, η^2^ = 0.022) and Alcohol Use (drinkers > non-drinkers; F = 3.988, *p* = 0.047, df = 209, η^2^ = 0.012), and a significant Time by Alcohol Use interaction (F = 9.180, *p* = 0.003, df = 209, η^2^ = 0.016, Figure 1). Post-hoc pairwise comparisons revealed that, whereas there was no difference in Negative Affect between drinkers and non-drinkers *before* the pandemic (t = 0.384, *p* = 0.71, df = 422), drinkers showed a significantly greater Negative Affect *during* the pandemic compared to non-drinkers (t = −3.89, *p* < 0.001, df = 422).

### 3.2. Change in Alcohol Use

The Wilcoxon signed-rank test showed that both drinkers and non-drinkers experienced a change in drinking frequency, such that drinkers reported a decrease (*Z* = −2.97, *p* = 0.003) and non-drinkers reported an increase (*Z* = −3.48, *p* < 0.001) in alcohol use frequency *during*, compared to *before*, the pandemic, though both groups remained on their same respective side of the alcohol use cut-off even after the change (i.e., at least once a month use of alcohol for drinkers, Table 2). Spearman correlations showed that among drinkers, more frequent alcohol use *before* the pandemic was marginally associated with a greater increase in negative affect during the pandemic (*r* = 0.237, *p* = 0.08). However, the associations between change in negative affect and both change in alcohol use (*r* = −0.188, *p* = 0.17) and frequency of alcohol use *during* the pandemic (*r* = −0.059, *p* = 0.67) were not statistically significant among drinkers.

### 3.3. Mediation of Social Isolation

**Figure 2:**
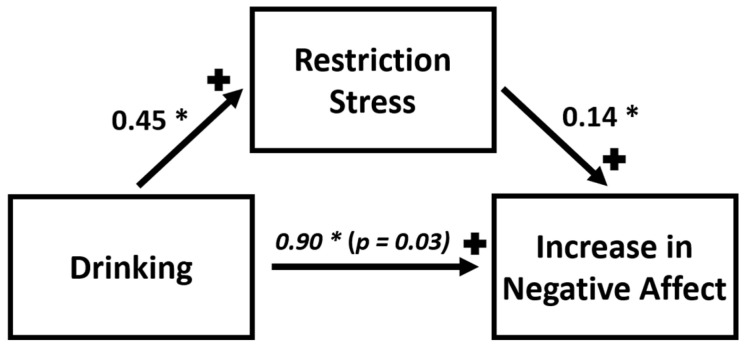
Stress from restrictions on leaving home was identified as a significant mediator of the positive correlation between early alcohol use (same between-group factor as used in the ANCOVA) and change in negative affect among participants.

Wilcoxon rank-sum comparisons demonstrated that, of the three questions related to the stress of social isolation, only the response to “How stressful were the restrictions on leaving home for you?” (restriction stress) significantly differed between groups (*Z* = −2.43, *p* = 0.02), (Table 2), whereas the responses to the other two items (social: *Z* = −1.61, *p* = 0.11; family-related: *Z* = −0.04, *p* = 0.97) did not reach statistical significance. Therefore, only ‘restriction stress’ was tested for possible mediation. The bootstrapped, unstandardized, indirect effect for restriction stress was 0.06 (0.45 * 0.14), with a mediated proportion of about 0.1 (1 − direct effect = 1 − 0.90) of the total effect of early drinking on change in negative affect (Figure 2).

### 3.4. Risk and Protective Factors

**Figure 3:**
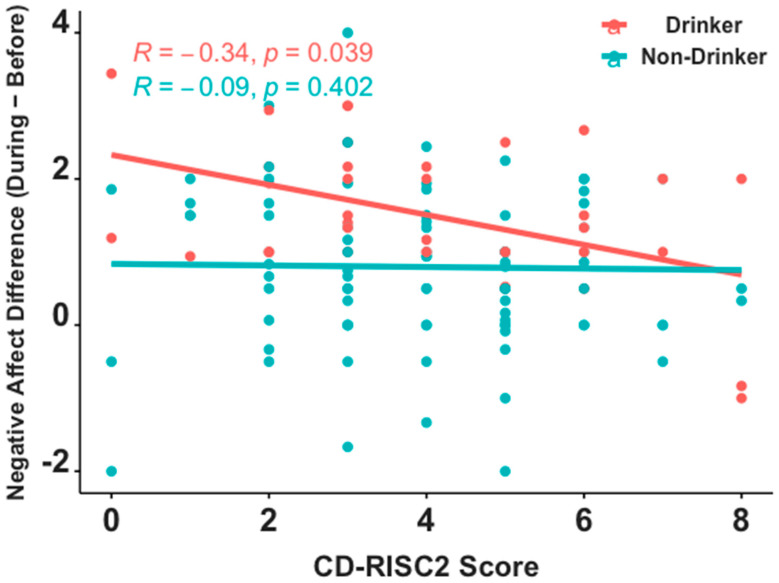
Spearman correlations on CD-RISC2 scores between young drinkers and non-drinkers. Higher CD-RISC2 scores signify higher levels of resilience.

**Figure 4:**
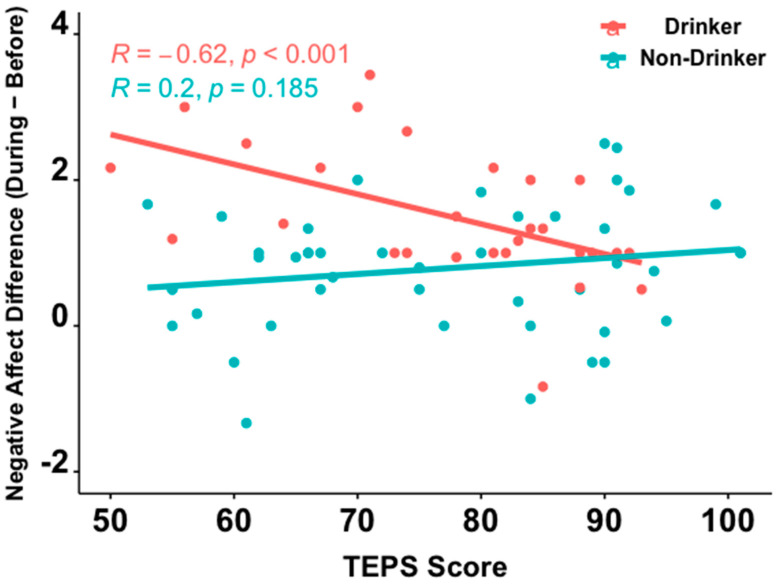
Spearman correlations on TEPS scores between young drinkers and non-drinkers. Higher TEPS scores signify lower levels of anhedonia.

Of the 217 participants included in our analysis, 127 subjects completed CD-RISC2 (38 (29.9%) drinkers, 89 (70.1%) non-drinkers), 103 completed S-UPPS-P (31 (30.1%) drinkers, 72 (69.9%) non-drinkers), and 73 completed TEPS (28 (38.4%) drinkers, 45 (61.6%) non-drinkers). Wilcoxon rank-sum tests showed no significant between-group differences in total scores on each of these three surveys (CD-RISC2: *Z* = −0.19, *p* = 0.85; S-UPPS-P: *Z* = −0.34, *p* = 0.74; TEPS: *Z* = −0.10, *p* = 0.92, Table 2). Across all participants, no statistically significant Spearman correlations were seen between Negative Affect and total CD-RISC2 (*r* = −0.169, *p* = 0.06), S-UPPS-P (*r* = −0.006, *p* = 0.96), or TEPS (*r* = −0.031, *p* = 0.79) scores. When examined separately within each group, lower resilience (i.e., higher CD-RISC2 scores) and higher anhedonia (i.e., higher TEPS scores) were associated with a greater increase in Negative Affect from *before* to *during* the pandemic in drinkers (CD-RISC2: *r* = −0.336; *p* = 0.039; TEPS; *r* = −0.623, *p* < 0.001), but not in non-drinkers (CD-RISC2: *r* = −0.090, *p* = 0.40; TEPS; *r* = 0.201, *p* = 0.19; Figure 3 and Figure 4). Between-group differences in these correlation coefficients were statistically significant for anhedonia (*Z* = −3.67, *p* < 0.001) and marginally significant for resilience (*Z* = −1.29, *p* = 0.099). The S-UPPS-P scores showed no significant association with a change in negative affect for either groups (drinkers: *r* = 0.009, *p* = 0.96; non-drinkers: *r* = −0.031, *p* = 0.79).

## 4. Discussion

Past research has found associations between negative affect, alcohol use, and stress in adolescents. However, few studies have investigated this relationship in the context of the COVID-19 pandemic, specifically to the unique stress of pandemic-related isolation. This is the first study, to our knowledge, that examined the impact of COVID-19 pandemic-related social restrictions on changes in negative affect in drinkers and non-drinkers, aged 21 and younger. We hypothesized that young drinkers would experience an exacerbated worsening of negative affect during the pandemic than non-drinkers. We tested whether youths that consumed alcohol regularly prior to the COVID-19 pandemic were more vulnerable to increases in negative affect, whether this relationship was mediated by the pandemic-related stress of social isolation, and whether factors such as resilience, impulsivity and anhedonia are associated with stress-induced changes in negative affect in young drinkers.

The primary, and expected, finding of this study is the worsening of negative affect from *before* to *during* the COVID-19 pandemic. This is consistent with prior literature demonstrating that daily stress may lead to increases in negative affect [38]. However, such an increase in negative affect from *before* to *during* the COVID-19 pandemic was, interestingly, more pronounced in young drinkers, compared to non-drinkers. The results revealed that while there was no significant difference in negative affect between drinkers and non-drinkers *before* the pandemic, drinkers reported a significantly greater increase in negative affect *during* the pandemic than non-drinkers. These findings are also supported by prior literature that suggests a positive relationship between underage alcohol use and negative affect [39]. Studies also suggest that environmental stressors are known risks for negative affect and underage drinking behavior [40]. Stress is also associated with both alcohol use and negative affect in adults [41].

Our study was unique in the way that the CRISIS questionnaire evaluated negative affect both *before* and *during* the COVID-19 pandemic and specifically inquired about the severity of perceived stress from pandemic-related social isolation. These data revealed that the stress induced by pandemic-related social isolation mediated the relationship between alcohol use and worsening negative affect. Although novel in its assessment of the influence of unique pandemic-related social isolation on the relationship between early drinking and an increase in negative affect, overall this finding is consistent with prior literature outlining social isolation as a stressor that poses risks to normative development in adolescents [42,43]. These results further suggest that such stressors may influence critical components of affect regulation [44], especially in younger drinkers. Our results also showed that greater alcohol use frequency *before* the pandemic was marginally associated with greater increase in negative affect among drinkers. Considering social distancing measures, which kept many young drinkers confined to the home, with limited access to alcohol, it is also not surprising that drinkers in our sample also experienced a significant decrease in frequency of alcohol use during the pandemic, although their alcohol use remained high enough during the pandemic for all of them to still qualify as drinkers, according to our pre-defined thresholds. However, such a decrease in alcohol consumption, a common social activity among young people, may have also contributed to their experienced heightened negative affect during COVID-19 lockdown. Such a relationship is consistent with past research on teenagers, which reported that a greater frequency in drinking occasions may alleviate emotional distress [45].

The findings that young drinkers with higher resilience and lower anhedonia showed less of an increase in negative affect, from *before* to *during* the pandemic, supports the hypothesis that higher resilience and lower anhedonia may serve as protective factors against a worsening of negative affect during stressful periods in young drinkers. This observation is consistent with literature examining the role of psychological factors as risk and protective forces at play in adjustment among adolescents [46]. It also expands on existing knowledge, connecting trait resilience [47] and anhedonia [48] to negative affect, by identifying functionally distinct associations of resilience and anhedonia with negative affect among young people, in the context of COVID-19. Other studies have shown similar associations between negative affect and anhedonia [49] and resilience [50], but particularly among adult drinkers. The lack of an association between impulsivity and increases in negative affect, from *before* to *during* the pandemic, in our data were not consistent with past literature suggesting that alcohol use may impact the influence of impulsivity on changes in negative affect among adolescents [51]. Given that impulsivity is a multifaceted construct, inconsistencies in these findings may arise from the use of different instruments to quantify impulsivity. For example, whereas we used the UPPS-P to assess trait impulsivity, Colder and Chassin (1997) used Eysenck’s impulsivity scale.

These results should be viewed in light of some potential limitations of this study that should be addressed in future research. First, this is a survey-based, cross-sectional study, in which questions regarding negative affect *before* and *during* the pandemic were asked at the same time (during the pandemic lockdown), which may have conflated the responses to these questions. Although a longitudinal study would have been ideal to study the effect of time (*before* versus *during*), this limitation is partially mitigated by having a comparison non-drinker group that also responded to the same questions during the same timeframe as the drinker group. Additionally, while limited in its generalizability, retrospective assessment employed by the CRISIS survey is a valid means of acquiring retrospective data. Past survey-based studies have commonly assessed substance use retrospectively among respondents, in the past 6–12 months [52,53]. Self-report surveys, such as PANAS-X (Positive Affect Negative Affect Schedule—Extended) that assess for ratings on negative emotions in the past year have also been validated and employed in studies on prior affective experiences [54,55].

Second, while the CRISIS survey included questions on alcohol use frequency, more information on alcohol use behaviors, including age of drinking onset, binge drinking, and alcohol use disorder diagnoses, would provide greater insight into the link between early drinking and negative affect during the COVID-19 lockdown. It is also worth considering that this study’s participants may have underreported their alcohol use, since it is likely that many of them are below the minimum legal drinking age. Additionally, there are certain language choices in the CRISIS survey that could have caused confusion among respondents. For example, one question in the survey, assessing educational level, includes “some grade school” as an answer choice. Though this term is meant to refer to all grades before high school, it is plausible that the ambiguity in this answer choice may have produced some uncertainty in participants [33]. Third, it is possible that other psychological factors may have contributed to the association between drinking and negative affect, beyond those that we selected based on priori literature (resilience, impulsivity, and anhedonia). Future studies should also include assessment of other factors, such as alexithymia, social functioning, and emotion regulation. Moreover, as adolescents continue to adapt to changing social contexts, it will be important to examine the clinical outcomes associated with increased stress-related negative affect during the COVID-19 pandemic. Special consideration should be given to exploring the role of dopamine, as extensive research has linked lower brain dopamine function to increased risk for behaviors such as alcohol use, which temporarily causes the neuronal release of dopamine [56]. Certain imaging studies have also found that a decrease in D2 receptors and mesolimbic dopamine transmission results in a hypodopaminergic state that can lead to alcohol use as a compensatory strategy [57,58]. Furthermore, factors explored in this study, such as anhedonia, have also been shown to be underpinned by the dopaminergic system and associated with negative affect and alcohol use [59]. Future studies should also explore the influences of trauma and socioeconomic conditions, considering past research has already indicated that adverse childhood experiences and economic disadvantage may predispose young people to underage drinking and harmful consequences associated with this activity [60,61]. Finally, the majority of our sample were female and received mental health diagnoses in the past. While both of these demographic factors were not found to serve as covariates, they indicate that our sample may not have been representative of the wider New York or United States population. Additionally, the high prevalence of mental health diagnoses in our sample specifically, may be attributed to the widespread negative impact of the COVID-19 pandemic on the mental health of young people, especially due to high positivity and fatality rates during the pandemic. Past research has also demonstrated that individuals with greater histories of psychiatric issues may feel more inclined to participate in mental health studies [62], possibly because of feelings of altruism or interest in the research itself [63]. Though mental health diagnosis was not significantly different between the two groups, it should be explored in future studies.

Taken together, these results are consistent with, and expand on, literature proposing multidimensional psychosocial risks associated with early drinking, with poor outcomes further exacerbated by stress and social isolation. It follows that COVID-19 may have served as a potent environmental stressor, leading to increased mental health concerns in young drinkers. Findings from this study lay the foundation for future research on protective and risk factors impacting mental health in vulnerable adolescents, especially during long-term social isolation. The significance of these results is further underscored by the interests of alcohol companies that have been shown to profit USD 17.5 billion a year from underage drinking, while their prevention strategies against this disturbing trend are negligible at best [64], possibly necessitating better policies to restrict the availability of alcohol to minors.

## 5. Conclusions

In this study we showed that young drinkers experienced a greater increase in negative affect during the COVID-19 pandemic compared to non-drinkers that was mediated by the pandemic-related stress of social isolation. However, this increase in negative affect among young drinkers was lower in those with greater resilience and lower anhedonia, which may have both served as protective factors against the worsening of negative affect in this group during the pandemic. Though these findings should be viewed in light of limitations in regard to data collection during the COVID-19 pandemic, they may inform future mental health studies on potential risk and protective factors in vulnerable young people during times of crisis.

## Figures and Tables

**Table 1:** Participant demographics and comparisons between alcohol users and non-users.

	Non-Drinkers		Drinkers		
	(*n* = 162)		(*n* = 58)		
Demographic Characteristic	*n*	%	*n*	%	Test Statistic
Age (years)					Z = −7.3 *
<18	101	62.3	7	12.1	
18–19	41	25.3	20	34.5	
20–21	20	12.3	31	53.4	
Sex					*X^2^* = 2.1
Male	33	20.4	13	22.4	
Female	118	72.8	43	74.1	
Other	11	6.8	1	1.7	
Race					*X^2^* = 2.8
White	113	69.8	47	81	
Black	5	3.1	1	1.7	
Other	44	27.2	10	17.2	
Hispanic	20	12.3	2	3.4	*X^2^* = 2.8
Education (highest level completed)					Z = −6.6 *
Some grade school	19	11.7	1	1.7	
Some high school	71	43.8	4	6.9	
High School or GED	27	16.7	11	19	
Some college or 2-year graduate	41	25.3	34	58.6	
4-year college graduate or higher	4	2.5	8	13.8	
Received mental health diagnosis	98	60.5	41	70.7	*X^2^* = 1.3
Marijuana use (at least once a month) ^a^	16	9.9	25	43.1	*X^2^* = 28.4 *
Tobacco use (at least once a month)	3	1.9	10	17.2	*X^2^* = 15.5 *

Z-values indicate Wilcoxon rank-sum test statistics, *X^2^* values indicate chi-squared test statistics, and asterisks (*) indicate statistical significance (*p* < 0.05) for between-group comparisons. ^a^ *n* = 218.

**Table 2:** Average scores and Wilcoxon tests of measures between alcohol users and non-users.

	Non-Drinkers	Drinkers	
Measure	Mean ± SD	Mean ± SD	Z
Negative affect score-*before* (from CRISIS)	2.70 ± 0.83	2.63 ± 0.83	−0.6
Negative affect score-*during* (from CRISIS)	3.51 ± 0.91	4.09 ± 0.81	−4.2 *
TEPS score (Anhedonia)	76.6 ± 14.4	77.3 ± 11.9	−0.1
CD-RISC2 score (Resilience)	4.01 ± 1.82	4.11 ± 2.09	−0.2
S-UPPS-P score (Impulsivity)	44.1 ± 8.97	45.2 ± 9.95	−0.3
Restrictions stress (from CRISIS)	3.02 ± 1.20	3.47 ± 1.21	−2.4 *
Loss of social contacts stress (from CRISIS)	3.04 ± 1.38	3.38 ± 1.34	−1.6
Loss of familial contacts stress (from CRISIS)	2.75 ± 1.35	2.75 ± 1.39	−0.04
Alcohol use frequency-*before* (from CRISIS)	1.22 ± 0.42	4.38 ± 1.24	−12.4 *
Alcohol use frequency-*during* (from CRISIS)	1.50 ± 1.14	3.60 ± 2.17	−8.0 *

The Z column indicates Z-values for Wilcoxon rank-sum tests and asterisks (*) indicate statistical significance (*p* < 0.05). Higher alcohol use scores indicate greater frequency in drinking.

## Data Availability

Data are available from the authors upon reasonable request.

## Abbreviations

COVID-19	Coronavirus disease of 2019;
REDCap	Research Electronic Data Capture
GAD	Generalized Anxiety Disorder
OCD	Obsessive-Compulsive Disorder
ADHD	Attention Deficit/Hyperactivity Disorder
CRISIS	Coronavirus Health Impact Survey
PANAS	Positive and Negative Affect Schedule
CD-RISC2	Connor-Davidson Resilience Scale (abbreviated version)
S-UPPS-P	Negative Urgency, Premeditation (lack of), Perseverance (lack of), Sensation Seeking, Positive Urgency, Impulsive Behavior Scale (abbreviated version)
TEPS	Temporal Experience of Pleasure
